# Lateral annular systolic excursion ratio: A novel measurement of right ventricular systolic function by two-dimensional echocardiography

**DOI:** 10.3389/fcvm.2022.971302

**Published:** 2022-09-02

**Authors:** Jonathan D. Stock, Eric S. Rothstein, Scott E. Friedman, Anthony S. Gemignani, Salvatore P. Costa, Andrew J. Milbridge, Rui Zhang, Cynthia C. Taub, Daniel J. O'Rourke, Robert T. Palac

**Affiliations:** ^1^Heart and Vascular Center, Dartmouth-Hitchcock Medical Center, Lebanon, NH, United States; ^2^Division of Cardiology, White River Junction VA Medical Center, White River Junction, VT, United States

**Keywords:** right ventricle (RV), right ventricular systolic function, RV function, cardiac magnetic resonance imaging, two-dimensional echocardiography, TAPSE, fractional area change

## Abstract

**Introduction:**

Accurate assessment of right ventricular (RV) systolic function has prognostic and therapeutic implications in many disease states. Echocardiography remains the most frequently deployed imaging modality for this purpose, but estimation of RV systolic function remains challenging. The purpose of this study was to evaluate the diagnostic performance of a novel measurement of RV systolic function called lateral annular systolic excursion ratio (LASER), which is the fractional shortening of the lateral tricuspid annulus to apex distance, compared to right ventricular ejection fraction (RVEF) derived by cardiac magnetic resonance imaging (CMR).

**Methods:**

A retrospective cohort of 78 consecutive patients who underwent clinically indicated CMR and transthoracic echocardiography within 30 days were identified from a database. Parameters of RV function measured included: tricuspid annular plane systolic excursion (TAPSE) by M-mode, tissue Doppler S', fractional area change (FAC) and LASER. These measurements were compared to RVEF derived by CMR using Pearson's correlation coefficients and receiver operating characteristic curves.

**Results:**

LASER was measurable in 75 (96%) of patients within the cohort. Right ventricular systolic dysfunction, by CMR measurement, was present in 37% (*n* = 29) of the population. LASER has moderate positive correlation with RVEF (r = 0.54) which was similar to FAC (r = 0.56), S' (r = 0.49) and TAPSE (r = 0.37). Receiver operating characteristic curves demonstrated that LASER (AUC = 0.865) outperformed fractional area change (AUC = 0.767), tissue Doppler S' (AUC = 0.744) and TAPSE (AUC = 0.645). A cohort derived dichotomous cutoff of 0.2 for LASER was shown to provide optimal diagnostic characteristics (sensitivity of 75%, specificity of 87% and accuracy of 83%) for identifying abnormal RV function. LASER had the highest sensitivity, accuracy, positive and negative predictive values among the parameters studied in the cohort.

**Conclusions:**

Within the study cohort, LASER was shown to have moderate positive correlation with RVEF derived by CMR and more favorable diagnostic performance for detecting RV systolic dysfunction compared to conventional echocardiographic parameters while being simple to obtain and less dependent on image quality than FAC and emerging techniques.

## Introduction

Accurate characterization of right ventricular (RV) systolic function has diagnostic and prognostic value in a variety of disease states including heart failure, pulmonary hypertension, chronic pulmonary disease, atrial fibrillation, valvular heart disease, congenital heart disease, pulmonary embolism and acute coronary syndrome (1–14). The imaging gold standard for assessing RV systolic function is volumetric analysis by cardiac magnetic resonance imaging (CMR) owing to its reproducibility and lack of reliance on geometric assumptions (15–19). However, transthoracic echocardiography (TTE) remains the most widely deployed imaging modality for this purpose due to its wide availability, portability and ease of use. The American Society of Echocardiography (ASE) has published guidelines for the assessment of RV function relying on qualitative and quantitative parameters using two-dimensional echocardiography, M-mode and tissue Doppler imaging with emerging roles of three-dimensional echocardiography and free wall longitudinal strain (20). Unfortunately, accurate assessment of RV function by TTE remains challenging owing to the RV's complex geometry, trabecular muscle structure and the difficulty in acquiring standardized imaging planes (21, 22).

Traditionally, the most commonly adopted parameters for measuring RV function have been M-mode derived tricuspid annular plane systolic excursion (TAPSE) and peak systolic annular velocity (S'). These measures are simple to obtain, reproducible and familiar to imagers due to decades of clinical use (20, 23–26). Despite these advantages, these one-dimensional parameters are highly angle dependent, may neglect radial contractile function, do not account for cardiac translational motion and correlate weakly with CMR derived right ventricular ejection fraction (RVEF) (15, 16, 20, 27, 28).

Two-dimensional fractional area change (FAC) offers a more comprehensive assessment of right ventricular systolic function by incorporating both longitudinal and radial contractile elements leading to improved diagnostic accuracy and correlation with CMR derived RVEF (27–29). However, FAC may suffer from foreshortening and interference from trabeculations. Accurate FAC measurement is highly dependent on the acquisition of a single imaging plane that visualized the base, free wall and apex of the RV.

Right ventricular free wall strain by speckle-tracking and RVEF by three-dimensional echocardiography are emerging techniques for the measurement of RV function. These techniques, though promising, rely an excellent image quality and have variable feasibility rates in inpatient and critical care settings (20, 30–38).

**Figure 1:**
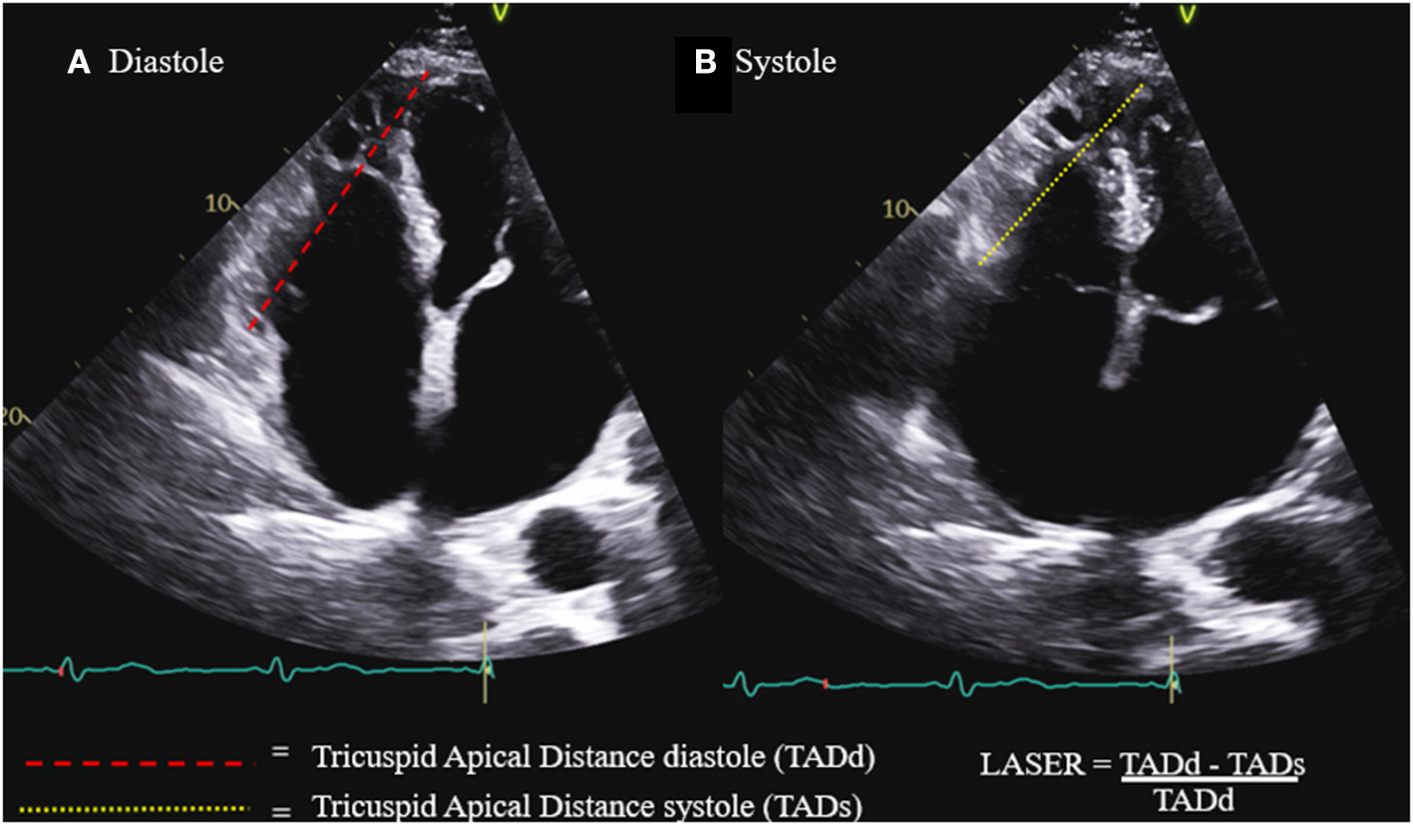
Lateral annular systolic excursion ratio (LASER) is the ratio of the systolic shortening of the tricuspid annulus to apex distance **(B)** compared to the length of the tricuspid annulus to apex distance in diastole **(A)**.

In clinical practice, there remains a need for a single parameter combining the simplicity and practicality of the one-dimensional parameters with the diagnostic accuracy of the more image quality dependent two and three-dimensional parameters. Lateral annular systolic excursion ratio (LASER) is a novel linear parameter which measures the fractional shortening of the linear distance between the lateral tricuspid annulus and the right ventricular apex (Figure 1). LASER is similar to TAPSE in that it incorporates the excursion of the lateral tricuspid annulus during systole, however, it improves upon the measurement by introducing an anchoring point at the RV apex which eliminates the angle dependence of the measurement and error associated with cardiac translation. Being a linear parameter with two anchoring points, LASER is less dependent on the acquisition of an optimal imaging plane and requires only the visualization of the lateral tricuspid annulus and the RV apex. Thus, LASER has the potential to be applicable across a diversity of patients and care settings.

The purpose of this study was to determine the correlation of LASER with RVEF derived by CMR and to determine the diagnostic ability of LASER for detecting abnormal RV function in comparison to TAPSE, S' and FAC.

## Materials and methods

### Study population

The study sample began with 163 consecutive adult patients who underwent CMR for any clinical indication between January 1, 2015 and December 31, 2018 at the White River Junction Veterans Affairs Medical Center in White River Junction, Vermont and at Dartmouth-Hitchcock Medical Center in Lebanon, New Hampshire. From this population, 82 patients were identified as having a TTE acquired within 30 days of CMR. Exclusion criteria were limited to patients who had a clinical event requiring hospitalization or urgent visit between the dates of the TTE and CMR or had a significant clinical change between studies one review of the health record, patients with irregular heart rhythms such as atrial fibrillation and patients who had otherwise uninterpretable short axis cine images on CMR making them unsuitable for volumetric analysis. Focused and technically limited echocardiograms including those with sub-optimal imaging windows were included in the study.

There were 78 patients who met the above inclusion criteria. Patient demographics, number of days between the CMR and TTE, and the indication for the CMR were collected by chart review. This study was approved by the local institutional review boards at both sites.

### Cardiac magnetic resonance imaging

CMR images were acquired using Siemens 1.5 Tesla whole body scanners (Siemens Healthineers, Erlangen, Germany) at both sites using a dedicated cardiac coil and electrocardiographic gating. Steady-state free precession cine images were acquired in short and long axis imaging planes. Right ventricular end-diastolic volumes (RVEDV) and right ventricular end-systolic volumes (RVESV) were quantitated in short-axis cine images (slice thickness 8 mm) with basal and apical image positions defined by the pulmonic valve annulus and the distal most myocardium respectively. The quantitation was accomplished according to a pre-specified analytic approach using commercially available software. Endocardial borders were measured by planimetry inclusive of trabeculae consistent with established standards. End-diastole and end-systole were defined by the largest and smallest cavity sizes, respectively.

### Echocardiography

Transthoracic echocardiograms were acquired by experienced sonographers using Philips Epiq, Philips iE33 (Philips Professional Healthcare, Amsterdam, Netherlands) and GE Vivid e95 ultrasound machines (GE Healthcare, Chicago, Illinois). Each examination included two-dimensional, M-mode, spectral Doppler and tissue Doppler imaging in the parasternal long axis (PLAX), parasternal short axis (PSAX), apical four chamber (A4C) and RV focused imaging planes as specified in the ASE guidelines (15). Some TTE examinations were clinical question focused and did not include M-mode and/or tissue Doppler imaging of the right ventricle.

Parameters of RV function were measured and calculated by two experienced readers (ER and RP) according to the ASE guidelines and included: TAPSE by M-mode, tissue Doppler S' and RV fractional area change (FAC). The measurement of LASER, as demonstrated in Figure 1, involves the identification of the lateral tricuspid annulus and drawing a line from this point to the endocardial tip of the RV apex. This line represents tricuspid annulus to apex distance (TAD). This distance is measured both in diastole and in systole. LASER is then calculated as the fractional shortening of this distance from diastole to systole: LASER = (TADd – TADs) / TADd.

### Reproducibility

Inter-observer and intra-observer reproducibility for each parameter of RV function including LASER were examined in a random sample of 17 patients. Intra-observer reproducibility was tested by a single reader (JS) and inter-observer reproducibility was tested between two readers (ER and JS). Readers were blinded to clinical history, CMR data and all previous measurements. All reproducibility measurements were acquired >30 days after initial measurements were made to reduce recall bias.

### Statistical analysis

Statistical analysis was performed using MedCalc statistical software version 18.6 (MedCalc Software, Ostend, Belgium). Each echocardiographic parameter of RV function including LASER was compared with CMR derived RVEF using correlation analysis to obtain Pearson's correlation coefficients. The cohort-derived diagnostic performance of each parameter for detecting abnormal RVEF, defined as < 50%, was determined by constructing receiver operating characteristic (ROC) curves (39). Area under the curve (AUC) was then used to rank the relative discriminatory strength of each parameter within the cohort using the Delong approach (40). An optimal cutoff to dichotomize abnormal and normal values for LASER was determined using Youden's index. The ASE recommended dichotomous cutoffs for abnormal TAPSE, S' and FAC were used for these parameters. The overall diagnostic accuracy for each parameter was compared using Fisher's exact test. Stepwise logistic regression analysis was performed to determine which parameters add the most predictive information in identifying abnormal RVEF. Inter-observer and intra-observer reproducibility was tested using Bland-Altman analysis as well as calculation of the mean relative difference between repetitive measurements (41).

## Results

### Sample characteristics

Baseline characteristics and CMR derived volumetric data for the cohort is displayed in Table 1. The cohort was predominantly male with a broad range of body mass indices represented. The mean time interval between the TTE and CMR was 8.7 days with a standard deviation of 9.2 days. RV function was variable with a mean ejection fraction of 49.7% with a standard deviation of 13.3% and range of 15–75%. The proportion of patients with abnormal RV function was 37%. Table 2 shows the diversity of clinical indications for CMR represented in the study cohort across 10 categories of indications.

**Table 1:** Patient characteristics and cardiac magnetic resonance imaging data of the sample population.

**Characteristics**	**Sample population (*n* = 78)**
Age (years)	58 ± 16 (18–83)
Male	58 (74%)
Body mass index (kg/m^2^)	29 ± 6.4 (14–49)
Time interval between imaging tests (days)	8.7 ± 9.2 (0–28)
Right ventricular end-diastolic volume index (mL/m^2^)	78.3 ± 24.7 (30–154)
Right ventricular ejection fraction (%)	49.7 ± 13.3 (15–75)
Normal right ventricular function	49 (63%)
Abnormal right ventricular function	29 (37%)
Left ventricular ejection fraction (%)	46 ± 18 (12–80)

Continuous variables are expressed as mean ± standard deviation (range) and proportions are expressed as number (percent).

**Table 2:** Diversity of clinical indications for cardiac magnetic resonance imaging by category.

**Indications**	**Quantity**
Valvular heart disease	8
Cardiac mass or thrombus	5
Congenital heart disease	3
Pericardial disease	5
Infiltrative cardiomyopathy	7
Hypertrophic cardiomyopathy	8
Non-ischemic cardiomyopathy	18
Myocarditis and sarcoidosis	9
Arrhythmia	5
Ischemic cardiomyopathy and viability	10

### Echocardiographic parameters compared to cardiac magnetic resonance imaging

LASER, FAC, tissue Doppler S' and TAPSE by M-mode were able to be measured in 75 (96%), 72 (92%), 53 (68%) and 58 (74%) patients within the cohort respectively. LASER was attainable in 96% of studies and FAC was attainable in 92% of the studies. Measurement of TAPSE and tissue Doppler S' were dependent upon requisite M-mode and tissue Doppler image acquisition which were not available in all patients. There were 44 (56%) patients within the cohort in whom all four parameters could be measured. The Pearson's correlation coefficient (r) for each parameter of RV function compared to CMR derived RVEF is shown in Table 3. FAC (r = 0.56) had the highest correlation followed by LASER (r = 0.54), tissue Doppler S' (r = 0.49) and TAPSE (r = 0.37).

**Table 3:** Pearson's correlation coefficients (r) for each echocardiographic parameter of right ventricular systolic function when compared to right ventricular ejection fraction (RVEF) by cardiac magnetic resonance imaging.

**Correlation of measurements with RVEF**			
**Measurement**	**n**	**r**	***p***
LASER	75	0.54	<0.001
Fractional area change	72	0.56	<0.001
Tissue Doppler S'	53	0.49	<0.001
TAPSE by M-mode	58	0.37	0.004

LASER, Lateral tricuspid annular systolic excursion ration; TAPSE, Tricuspid annular plane systolic excursion.

**Figure 2:**
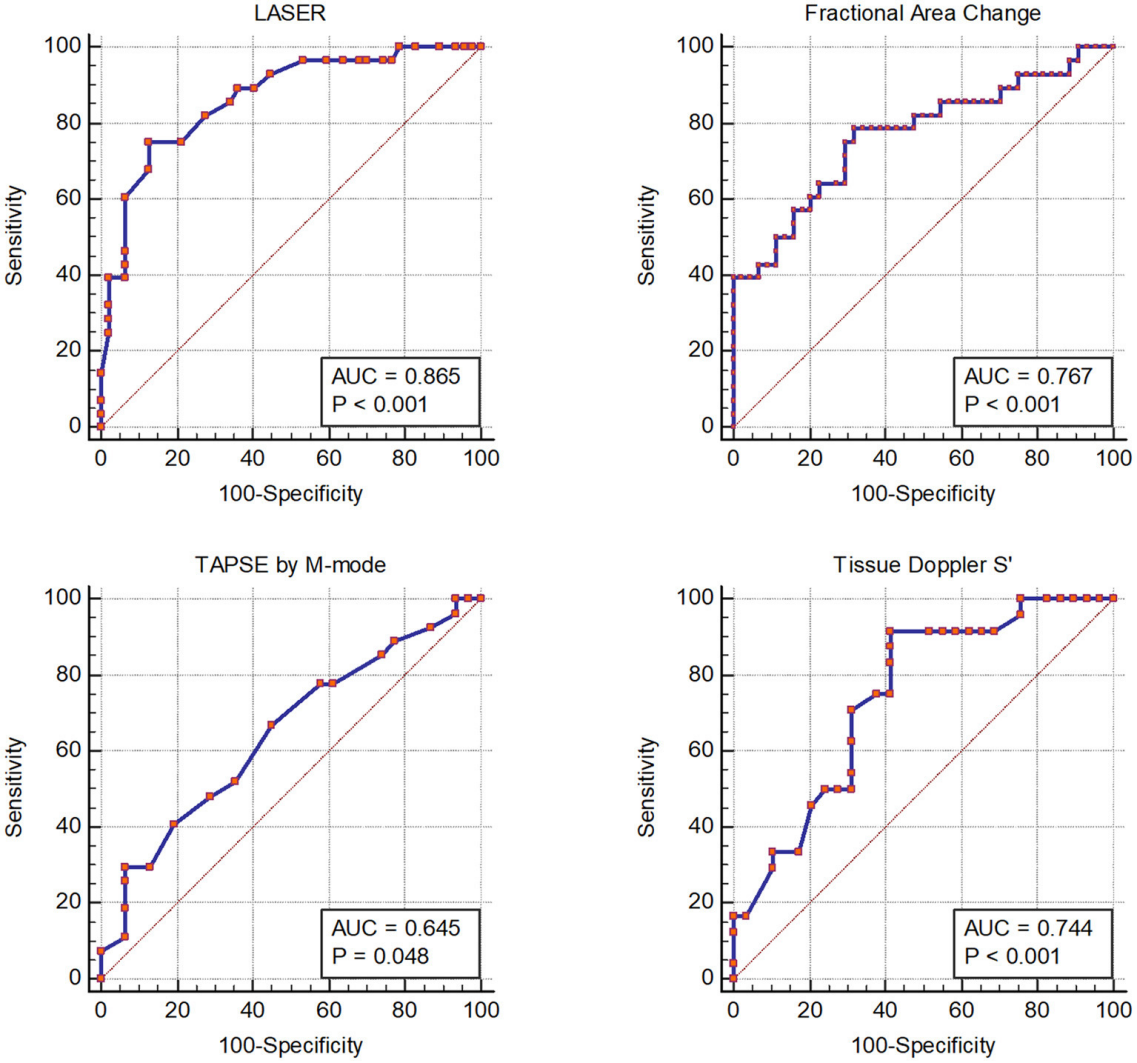
Receiver operator characteristic curves showing the diagnostic performance of each echocardiographic parameter of right ventricular systolic function compared to right ventricular ejection fraction by cardiac magnetic resonance imaging.

**Figure 3:**
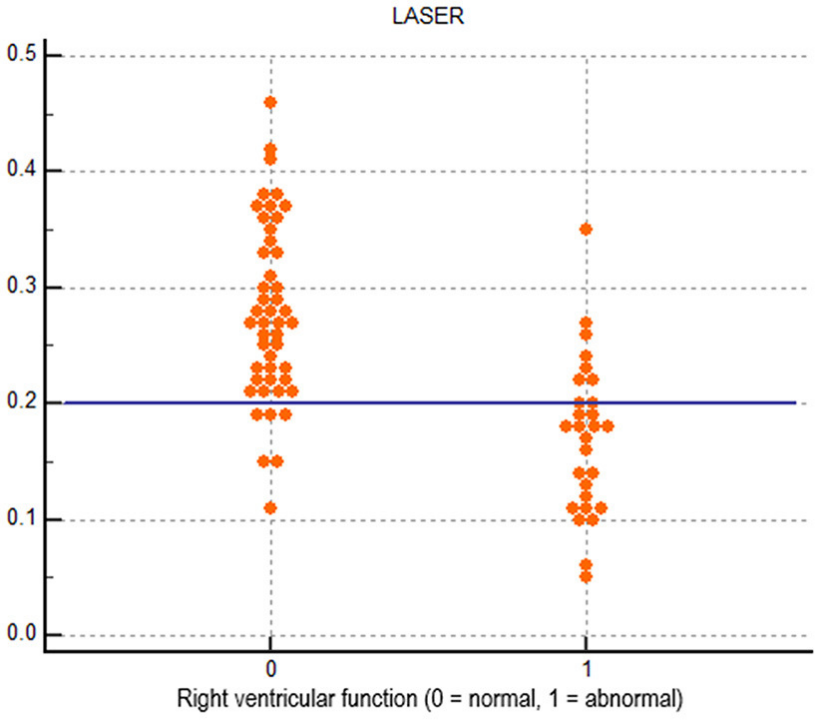
Dot diagram showing the diagnostic performance of the dichotomous cutoff of 0.2 for lateral tricuspid annular systolic excursion ratio (LASER).

Receiver operating characteristic curves for each echocardiographic parameter of RV function compared to CMR derived RVEF are displayed in Figure 2. LASER (AUC = 0.865) had the highest diagnostic ability for detecting abnormal RVEF followed by FAC (AUC = 0.767), tissue Doppler S' (AUC = 0.744) and TAPSE (AUC = 0.645). The optimal dichotomous cutoff value between normal and abnormal LASER was determined to be 0.2 with a Youden's Index of 0.62 and an associated sensitivity and specificity of 75% and 87% respectively (Figure 3).

Table 4 displays the cohort derived diagnostic performance of each parameter for detecting abnormal RVEF using the cohort derived cutoff value for LASER (0.2) and cutoff values recommended by the American Society of Echocardiography for FAC (35%), tissue Doppler S' (9.5 cm/s) and TAPSE (17 mm). LASER had the highest sensitivity (75%), accuracy (83%), positive predictive value (78%) and negative predictive value (85%). LASER's diagnostic accuracy was statistically comparable to that of FAC (*p* = 0.231) by Fisher's exact test. The diagnostic accuracy of LASER was significantly higher than tissue Doppler S' (*p* = 0.008) and TAPSE (*p* = 0.006).

**Table 4:** Cohort derived test characteristics for each echocardiographic parameter of right ventricular systolic function.

**Diagnostic performance of right ventricular measurements**							
**Measurement**	***n***	**Prevalence**	**Sensitivity**	**Specificity**	**Accuracy**	**PPV**	**NPV**
LASER	75	37%	75%	87%	83%	78%	85%
Fractional area change	72	39%	50%	89%	74%^*^	74%	74%
Tissue Doppler S'	53	45%	33%	83%	61%^†^	62%	60%
TAPSE by M-mode	58	47%	48%	71%	60%^‡^	59%	61%

Prevalence represents the prevalence of abnormal right ventricular ejection fractions within each parameter's sample.

LASER, Lateral annular systolic excursion ratio; TAPSE, Tricuspid annular plane systolic excursion; PPV, Positive predictive value; NPV, Negative predictive value.

* Statistically insignificant difference compared to LASER p = 0.231 (Fisher's Exact).

† Statistically significant difference compared to LASER p = 0.008 (Fisher's Exact).

‡ Statistically significant difference compared to LASER p = 0.006 (Fisher's Exact).

Stepwise logistic regression analysis was performed using the patients in which all parameters were measure (*n* = 44). The model showed most predictive parameters for abnormal RVEF in our cohort were LASER (*p* = 0.004) and S' (0.030) with LASER being the most important component of the model. FAC and TAPSE did not add additional value beyond LASER or S' in the model.

### Reproducibility

Table 5 shows the inter-observer and intra-observer reproducibility for the measurement of each parameter of RV function. The traditional 1-dimensional parameters of S' and TAPSE proved to be most reproducible with mean relative differences between observers of 1.3 and 3.4% respectively. LASER and FAC were slightly less reproducible with mean relative differences of 4.2 and 5.4% respectively.

**Table 5:** Inter-observer and intra-observer reproducibility of measurements.

**Methodologic reproducibility**
	**Inter-observer reproducibility**			**Intra-observer reproducibility**		
**Measurement**	**Mean difference** **±standard deviation**	**Mean relative difference (%)**	**95% limits of agreement**	**Mean difference** **±standard deviation**	**Mean relative difference (%)**	**95% limits of agreement**
LASER	0.02 ± 0.04	4.2	−0.06 to 0.09	0.02 ± 0.03	4.2	−0.04 to 0.09
FAC	4.7 ± 6.7	5.4	−8.5 to 18.0	−0.15 ± 7.8	4.4	−1.5 to 1.5
S' (cm/s)	0.00 ± 0.87	1.3	−1.7 to 1.7	0.00 ± 0.61	0.4	−1.2 to 1.2
TAPSE (mm)	−0.05 ± 3.2	3.4	−0.68 to 0.57	−0.06 ± 0.17	2.1	−0.39 to 0.28

LASER, lateral annular systolic excursion ratio; TAPSE, tricuspid annular plane systolic excursion; FAC, fractional area change.

## Discussion

This is the first study evaluating the correlation and diagnostic performance of the novel measurement of RV function LASER in a broad cohort of patients with a high prevalence of abnormal RV function. Measurement of LASER was achievable in 96% of patients despite many of the echocardiograms being technically limited or problem focused. These data suggest LASER is attainable across a variety clinical care settings such as the emergency department, critical care unit or cardiac catheterization laboratory where optimal image quality may be difficult to obtain or when the scanner is not a registered diagnostic cardiac sonographer.

The LASER technique has moderate positive correlation (r = 0.54) with CMR derived RVEF, comparable to that of FAC (r = 0.56) while the one-dimensional parameters of tissue Doppler S' (r = 0.49) and TAPSE (r = 0.37) had fair correlation. The correlation of FAC with RVEF in this study was similar to that in larger cohorts with Kim et al. reporting a correlation coefficient of 0.55 in 272 patients with coronary artery disease and Pavlicek et al. reporting 0.472 in 223 patients (15, 16). The correlation of TAPSE and S' with RVEF has been variable and weak in large cohorts. Kim et al. reported correlation coefficients for TAPSE and S' of 0.48 and 0.36 respectively, whereas Pavlicek et al. reported 0.336 and 0.476 (15, 16). The correlations of TAPSE and S' with RVEF in this cohort were similar to the prior values reported by the aforementioned authors.

The diagnostic ability of LASER to detect abnormal RV function was favorable using receiver operating characteristic curves with an AUC of 0.865 which was larger than that of FAC (0.767), TAPSE (0.645) and S' (0.744). Using the cohort derived cutoff of 0.2, LASER had the highest sensitivity, accuracy, positive predictive value and negative predictive value among the studied parameters of RV function using ASE recommended cutoffs. The diagnostic accuracy of LASER was 83% which was statistically similar to FAC (74%) with a p value of 0.231 and better than TAPSE (60%) and S' (61%) with p values of 0.008 and 0.006 respectively. The diagnostic performance of FAC, TAPSE and S' in this cohort was similar to the diagnostic performance reported by Pavlicek et al. with AUCs of 0.728, 0.716 and 0.779 for each parameter respectively (16). Agasthi et al. in a large cohort of 500 patients reported a lower diagnostic performance of FAC, TAPSE and S' with AUCs of 0.6658, 0.5819 and 0.5909 respectively, though a different dichotomous cutoff for abnormal RVEF was used (28). The stepwise logistic regression model suggests that FAC and TAPSE do not add additional diagnostic value over LASER and S' which further suggests that LASER as a simple measure may be a robust parameter for discriminating abnormal RV function.

Overall, this study shows LASER is a simple to acquire, robust and reproducible measurement of RV function with similar diagnostic performance to FAC and better diagnostic performance than the traditional one-dimensional parameters of TAPSE and S'. The main advantage of LASER over FAC is that it can be performed in most patients even when image quality is poor as it requires only the visualization of the lateral tricuspid annulus and RV apex. Although not evaluated in this study, theoretically this also translates to RV free wall strain and RVEF by 3DE as these measurements also require good image quality with perfect imaging windows for visualization of the entire RV free wall throughout the cardiac cycle. A possible niche use for LASER may indeed be in the emergency department, critical care unit or cardiac catheterization laboratory where a rapid assessment of RV function is often necessary for clinical decision making and perfect imaging planes may be difficult to acquire.

### Study limitations and future directions

Though the study cohort included a broad diversity of pathology and a high prevalence of abnormal RV function, the size of the cohort was relatively small at 78 patients and included mostly men. The echocardiograms included were not acquired for the purpose of studying RV function nor was a single standardized protocol used for each echocardiogram. Rather, images were acquired following different acquisition protocols depending on the indication for the study with focused echocardiograms and stress echocardiograms being included in the cohort. One could argue that this may better represent “real-world” images in a busy clinical practice, but it also limits the yield of useful data as many of the included patients lacked the requisite tissue Doppler or M-mode image acquisitions for the measurement of S' and TAPSE reducing the sample size for these measurements. This limited sample size may reduce the certainty of the comparisons and explain some of the variability in the diagnostic performance of these parameters compared to larger cohorts with images acquired using a standardized research protocol. However, the calculated AUC for S' and TAPSE were similar to those reported in larger studies (15, 16).

Another limitation related to the retrospective nature of the dataset is the time interval range between the acquisition of CMR images and TTE images. Acquiring the CMR and TTE images on different days introduces the possibility of different preload and afterload conditions which may influence RVEF and introduce unaccounted for variation between the TTE derived functional parameters and CMR derived RVEF (42), although the study team did its best to exclude any patients where clear clinical changes occurred.

It is important to note that this cutoff of 0.2 was derived from our cohort and not from a healthy volunteer population and further studies in either healthy volunteer populations or with larger proportions of normally functioning right ventricles will be necessary to fully understand cutoff values.

LASER is the sum of both the radial and longitudinal vectors of right ventricular motion, and in this study we were unable to determine the precise contribution of each vector. If the longitudinal vector contributes significantly more than the radial vector, it is certainly possible that the sensitivity of LASER may be impaired in conditions that primarily induce radial dysfunction, such as some cases of pulmonary hypertension. Furthermore, we were also unable to use our data set to evaluate the performance of LASER with different RV shapes. An understanding of the strengths and limitations of LASER as a measurement of RV function would ideally be addressed in a future CMR based study that would be able to evaluate LASER with optimal visualization of the free wall and without interference from trabeculations.

Additionally, RV Free wall strain and 3D RV functional assessment were not performed, as these software packages were not available in the echocardiography laboratories during the study period.

Lastly, the chosen dichotomous cutoff value for abnormal RVEF was <50% in this study as it was similar to two other large cohorts used to assess parameters of RV structure and function (15, 16). Other studies have used an RVEF <45% to dichotomize normal from abnormal (27–29). The optimal cutoff value for defining abnormal RVEF is not known with some authors advocating for age, sex and BMI adjusted cutoffs (17).

Given the above limitations, further studies are required to verify the diagnostic performance of LASER in larger, more diverse cohorts prior to deployment of the measure in clinical practice. Disease specific outcome data and correlation with right ventricular systolic pressure will also establish the usefulness of this simple linear measure acquired by two-dimensional echocardiography.

## Conclusion

This study demonstrates that LASER, a novel, easy to measure parameter of RV systolic function, has moderate correlation with RVEF derived by CMR and diagnostic accuracy comparable to FAC and superior to TAPSE and S'. The advantage of LASER is that it is less reliant on image quality and optimal imaging planes compared to other parameters such as FAC, free wall strain and RVEF by 3DE making it potentially suitable for deployment in a wide range of clinical care settings.

## Data availability statement

The raw data supporting the conclusions of this article will be made available by the authors, without undue reservation.

## Ethics statement

The study was approved by the internal review boards at the White River Junction VA Medical Center and Dartmouth-Hitchcock Medical Center. Ethical review and approval/written informed consent was not required as per local legislation and institutional requirements.

## Author contributions

ER, JS, SF, and RP conceived the study, formulated hypotheses, and collected and analyzed the data. JS, ER, and RP performed the statistical analysis. JS and ER prepared the manuscript. AM, AG, DO'R, RZ, and RP critically evaluated the data and edited the manuscript. All authors read and approved the final manuscript and agreed to be accountable for all aspects of the work.

## Conflict of interest

The authors declare that the research was conducted in the absence of any commercial or financial relationships that could be construed as a potential conflict of interest.

## Publisher's note

All claims expressed in this article are solely those of the authors and do not necessarily represent those of their affiliated organizations, or those of the publisher, the editors and the reviewers. Any product that may be evaluated in this article, or claim that may be made by its manufacturer, is not guaranteed or endorsed by the publisher.

## Abbreviations

RV: Right Ventricle
CMR: Cardiac Magnetic Resonance Imaging
TTE: Transthoracic Echocardiogram
ASE: American Society of Echocardiography
TAPSE: Tricuspid Annular Plane Systolic Excursion
RVEF: Right Ventricular Ejection Fraction
FAC: Fractional Area Change
LASER: Lateral Annular Systolic Excursion Ratio
RVEDV: Right Ventricular End-Diastolic Volume
RVESV: Right Ventricular End-Systolic Volume.
